# High Diversity of Hepatitis B Virus Genotypes in Panamanian Blood Donors: A Molecular Analysis of New Variants

**DOI:** 10.1371/journal.pone.0103545

**Published:** 2014-08-05

**Authors:** Alexander A. Martínez, Yamitzel Y. Zaldivar, CSS-NAT Group, Zoila De Castillo, Alma Y. Ortiz, Yaxelis Mendoza, Juan Cristina, Juan M. Pascale

**Affiliations:** 1 Gorgas Memorial Institute for Health Studies, Panama, Panama; 2 Department of Biotechnology, Acharya Nagarjuna University, Guntur, A.P. India; 3 INDICASAT-AIP Clayton, City of Knowledge, Panama; 4 Complejo Hospitalario Dr. Arnulfo Arias Madrid, Caja de Seguro Social, Panama, Panama; 5 Laboratorio de Virología Molecular, Centro de Investigaciones Nucleares, Facultad de Ciencias, Universidad de la República, Montevideo, Uruguay; CRCL-INSERM, France

## Abstract

Hepatitis B Virus (HBV) is an infectious agent that causes more than half of the cases of liver disease and cancer in the world. Globally there are around 250 million people chronically infected with this virus. Despite 16% of the cases of liver disease in Central America are caused by HBV, the information regarding its genetic diversity, genotypes and circulation is scarce. The purpose of this study was to evaluate the genetic variability of the HBV genotypes from HBV-DNA positive samples obtained from screening blood donors at the Social Security System of Panama and to estimate its possible origin. From 59,696 blood donors tested for HBV infection during 2010–2012, there were 74 HBV-DNA positive subjects. Analysis of the partial PreS2-S region of 27 sequences shows that 21% of the infections were caused by genotype A, 3% by genotype D and 76% by genotype F. In addition, we were able to confirm circulation of six sub-genotypes A1, A2, A3, D4, F3, F1 and a proposed new sub-genotype denominated F5pan. We found a confinement of sub-genotypes F1 and F5pan to the western area of Panama. The tMRCA analysis suggests a simultaneous circulation of previously described sub-genotypes rather than recent introductions of the Panamanian sub-genotypes in the country. Moreover, these results highlight the need of more intensive research of the HBV strains circulating in the region at the molecular level. In conclusion, Panama has a high HBV genotype diversity that includes a new proposed sub-genotype, an elevated number of PreCore-Core mutations, and confinement of these variants in a specific geographical location.

## Introduction

Hepatitis B Virus (HBV) is an infectious agent that causes more than half of the cases of liver disease and cancer in the world [1]. Globally there are around 250 million people chronically infected with this virus [2], in Latin America, the HBV prevalence levels go from low (<2%) to intermediate (2% to 8%) depending on the population analyzed [3]. In this region, the virus is also the etiologic agent of around 14% of the cases of hepatocellular carcinoma (HCC) [4]. HBV can be classified in nine genotypes (5–9), according to a nucleotide intergroup divergence greater than 8% of the whole genome, and in twenty sub-genotypes, if a nucleotide intergroup divergence of 4% to 7.5% is considered [5], [6]. The study of the HBV in several clinical settings showed important differences between genotypes in the observed clinical outcomes [7]. Besides, the study of sub-genotypes allowed a more accurate determination of HBV geographical distribution patterns, an example, is the recent estimation of the migration path of the HBV genotype A1 in and out of Africa [8].

Information regarding the genetic diversity of the HBV in Central America is scarce. The most concise reports of the HBV Central American diversity were conducted 15 years ago [9], [10]. Moreover, previous studies describing the HBV diversity were done using partial, discrete regions of the HBV genome [11]. Another evidence of the scarce number of studies of HBV in the region is the small number of whole genome sequences of Central American origin, deposited in international databases (n<100) [12].

In Panama, HBV diversity was first described in the Chinese population residing in the metropolitan area. This study showed that only genotypes from Asia (Genotypes B and C) are circulating in this group [13]. Still, there is a lack in the knowledge regarding the diversity and molecular characteristics of this virus in the country. Since 2010, a centralized continuous screening of blood donors by molecular techniques: Nucleic Acid Test (NAT) has been implemented in Panama. The use of this technique created a great opportunity to evaluate the circulating HBV genotypes in blood donors from different regions of the country. The purpose of this study was to evaluate the genetic diversity of the HBV genotypes from HBV-DNA positive samples obtained from screening blood donors at the Social Security System of Panama from 2010 to 2012. In addition, we estimated the possible regional origin of these genotypes.

## Methods

### Ethics Statement

The study cohort included positive samples analyzed anonymously from selected blood donors who were screened from the Social Security Health System of Panama. The study protocol was submitted and approved by The Gorgas Memorial Institutional Ethics Review Board.

### Population studied

The Panamanian Social Security Health System has a country network of blood banks that receives approximately 20,000 blood donors annually. Plasma samples from blood donors from seven cities of Panama (Colon, Panama City, Panama Oeste, Penonome, Los Santos, Chiriqui, and Changuinola) are sent for HBV-DNA screening at the centralized nucleic acid test (NAT) unit of the Complejo Hospitalario Dr. Arnulfo Arias Madrid (CHDrAAM). Samples were collected during the period of January, 2010 to December, 2012. All HBV positive samples were included in this study if they fulfilled the following requirements: no less than 300 µL of plasma and storage at −80°C.

### Nucleic acid test

Blood donors were screened for HBV-DNA using pools of six samples for a total volume of 1 mL. Each pool was evaluated using the Cobas TaqScreen MPX Test (Roche Molecular Systems, USA). Reactive pools were tested individually to define the positive samples.

### DNA Extraction and PCR amplification

DNA was extracted from 300 µl of plasma using commercial DNA/RNA extraction kits (Qiagen, USA). The presence of HBV-DNA was confirmed and quantified using a previously described method [14]. For the preliminary genotyping, samples with detectable viremia were subjected to a nested PCR using a high proof reading enzyme mixture (Platinum PCR Super Mix High Fidelity, LifeTech, USA), that yielded a 879 bp fragment. Sequencing was performed in an automated DNA sequencer (ABI 3130xl, LifeTech, USA) using primers previously described [13].

The full-length genome of Panamanian sequences clustering in particular clades of genotype F, were amplified using two protocols previously published [15], [16]. When a sample failed to yield a good quality product with the one-step protocol, the nested approach was used.

The sequences obtained in this study were deposited in Genbank under the accession numbers KJ638656-KJ638679.

### Genotype determination by phylogenetic analysis

Hepatitis B genotypes were determined comparing published reference sequences (n = 281) with the PCR nested product (879 bp) obtained from 27 Panamanian sequences. The HBV sequences were aligned with Muscle v3.8.31 [17] and manually inspected, the final dataset is available upon request. The phylogenetic tree was inferred under a Maximum Likelihood (ML) method and the nucleotide substitution model (GTR+I+Γ) was selected using the jModeltest2 program [18]. The ML tree was reconstructed with the PhyML program using an online web server [19]. A heuristic tree search was performed using the SPR branch-swapping algorithm and the reliability of the obtained topology was estimated with the approximate likelihood-ratio test (αLTR) based on the Shimodaira-Hasegawa-like procedure [20]. The ML trees were visualized using MEGA v.5.2 [21].

### Sub-genotype Analysis

Recombination analysis of the whole genome sequences was performed in RDP4 [22] software using RDP, MAXCHI, CHIMAERA, BOOTSCAN, as exploratory methods, with a window size of 60, 120, 500, 500, respectively.

The phylogenetic analysis of the whole genome, PreS2-S and PreC-Core gene was performed separately. The best-fit nucleotide substitution model (GTR+I+Γ for whole genome and PreS2-S; HYK+I+Γ for PreC-Core gene) was determined with Jmodeltest2 [18], and the ML tree was drawn as above, the reliability of the tree topology was also calculated by bootstrapping (1000 replicates).

The genetic distances among HBV genotype F sequences obtained and sub-genotype F reference sequences (n = 68) were calculated using p-distances, standard error estimated were calculated by bootstrapping (1000 replicates). Evolutionary analysis was conducted in MEGA 5.2 [21].

Amino acid substitutions in polymerase, PreS2-S, HBsAg and X-gene were determined by translating the Panamanian nucleotide sequences and comparing them against reference sub-genotype F amino acid sequences [16], [23], using MEGA 5.2 [21].

### Bayesian analysis

Full-length genome and partial polymerase (nt 177 to 990) gene datasets were constructed including published reference sequences for sub-genotypes of genotype F [16]. An uncorrelated lognormal relaxed clock (UCLN) [24] was used to evaluate the time of the most recent common ancestor (tMRCA) of each node. Sample collection date was used as a calibration to calculate the substitution rates; additionally, a fixed medium rate of 1.5×10^−5^ [16] was used to calculate the tMRCA of each node. The best-fit demographic tree model was selected calculating the path sampling (PS) and stepping stone sampling methods [25]. MCMC chains were run in BEAST v1.8 [26] for 50×10^6^ generation for the polymerase datasets and for the complete genome dataset. Convergence of chains was estimated by calculating the effective sample size (>200), and uncertainty in parameter estimates with the 95% highest probability density (HPD), all values were calculated after excluding an initial 10% of burn-in using TRACER V1.6.

The tMRCA of the whole HBV genome of the Panamanian samples was calculated using a partitioned model [27], in which, the genome was partitioned in the corresponding coding genes and overlapping regions, a different substitution model is applied to each partition (Table S1) [27].

## Results

### Low prevalence of HBV-DNA and HBeAg in blood donors

During the period of 2010 to 2012, 59,697 blood donors samples were analyzed at the NAT unit in the Complejo Hospitalario Dr. Arnulfo Arias Madrid (CHDrAAM), and 79 (0.13%) of them were HBV-DNA positive. A total of 57 (72%) positive HBV-DNA samples were included in this study, 33 of these samples had HBV-DNA level greater than 500 copies/ml (mean = 1.15×10^7^, standard error of mean = 7.8×10^6^). Twenty-seven of them were successfully sequenced and used for the phylogenetic analysis of partial PreS2-S region.

### High genetic diversity of Hepatitis B virus genotypes in Panamanian blood donors

**Figure 1:**
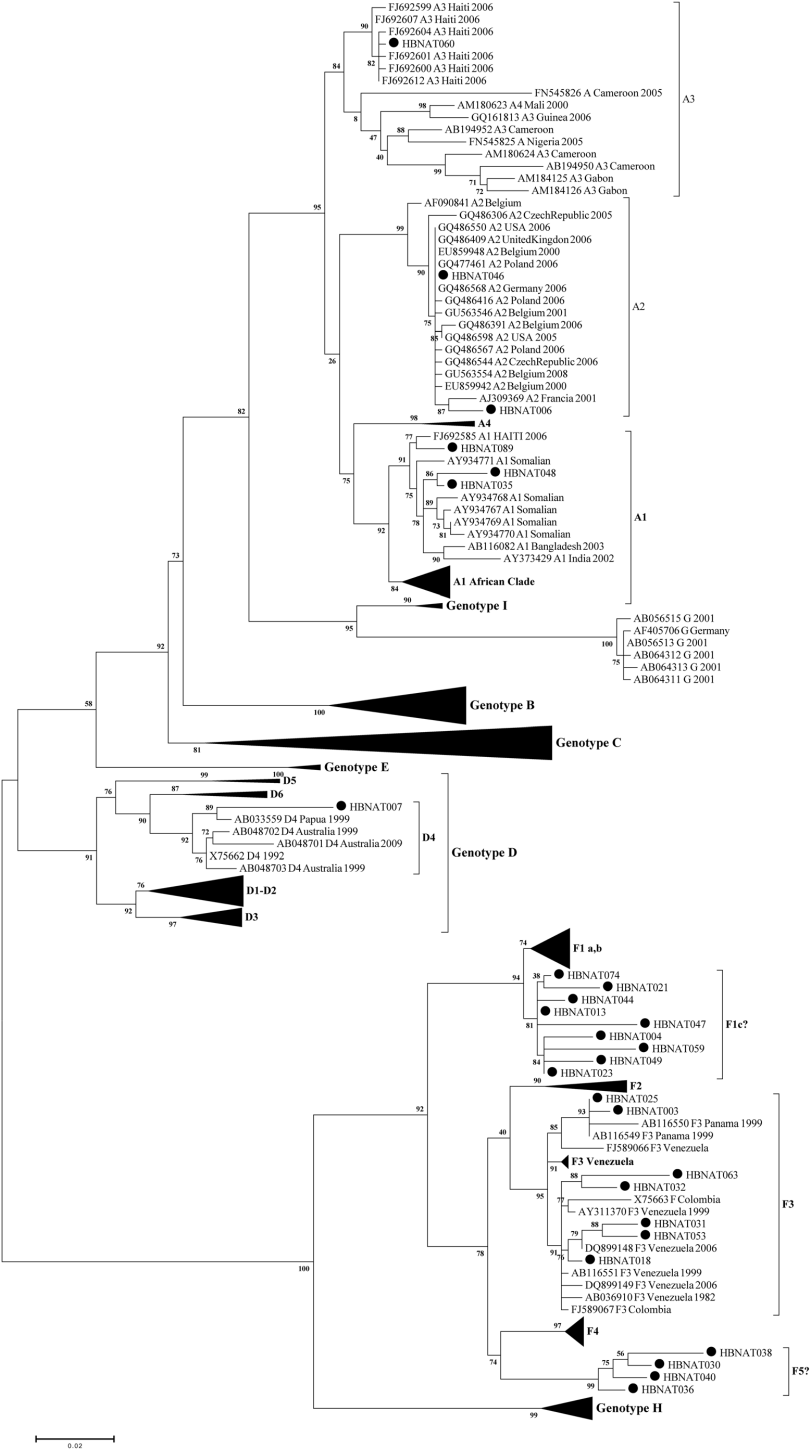
Midpoint rooted Maximum Likelihood tree based on HBV partial PreS2-S reference sequences of each genotype (A–I) (n = 280) and sequences of Panamanian origin. (∼820 pb, n = 27, bold circle). Clades in which there are not Panamanian samples are collapsed to simplify, node numbers correspond to αLTR values higher than 0.50.

The phylogenetic analysis of partial PreS2-S region showed that the prevalent circulating HBV genotypes in Panama are: A (n = 6), D (n = 1), and F (n = 20) (Fig. 1). For Genotype A, sub-genotypes A1, A2 and A3 were found in 3 (11%), 2 (7.5%) and 1 (3.7%) samples, respectively. For the genotype F, 9 (33%) sequences were closely related to the branch of sub-genotype F1, but in a monophyletic clade separated from the previous reported F1a, F1b. Other groups of sequences (n = 7, 25.9%) were sub-genotype F3, and four sequences (14%) fell within the genotype F branch, but form a new monophyletic cluster of sub-genotype F (Fig. 1).

### New variants of Sub-genotypes of HBV genotype F are circulating in blood donors, in a region of the country

**Figure 2:**
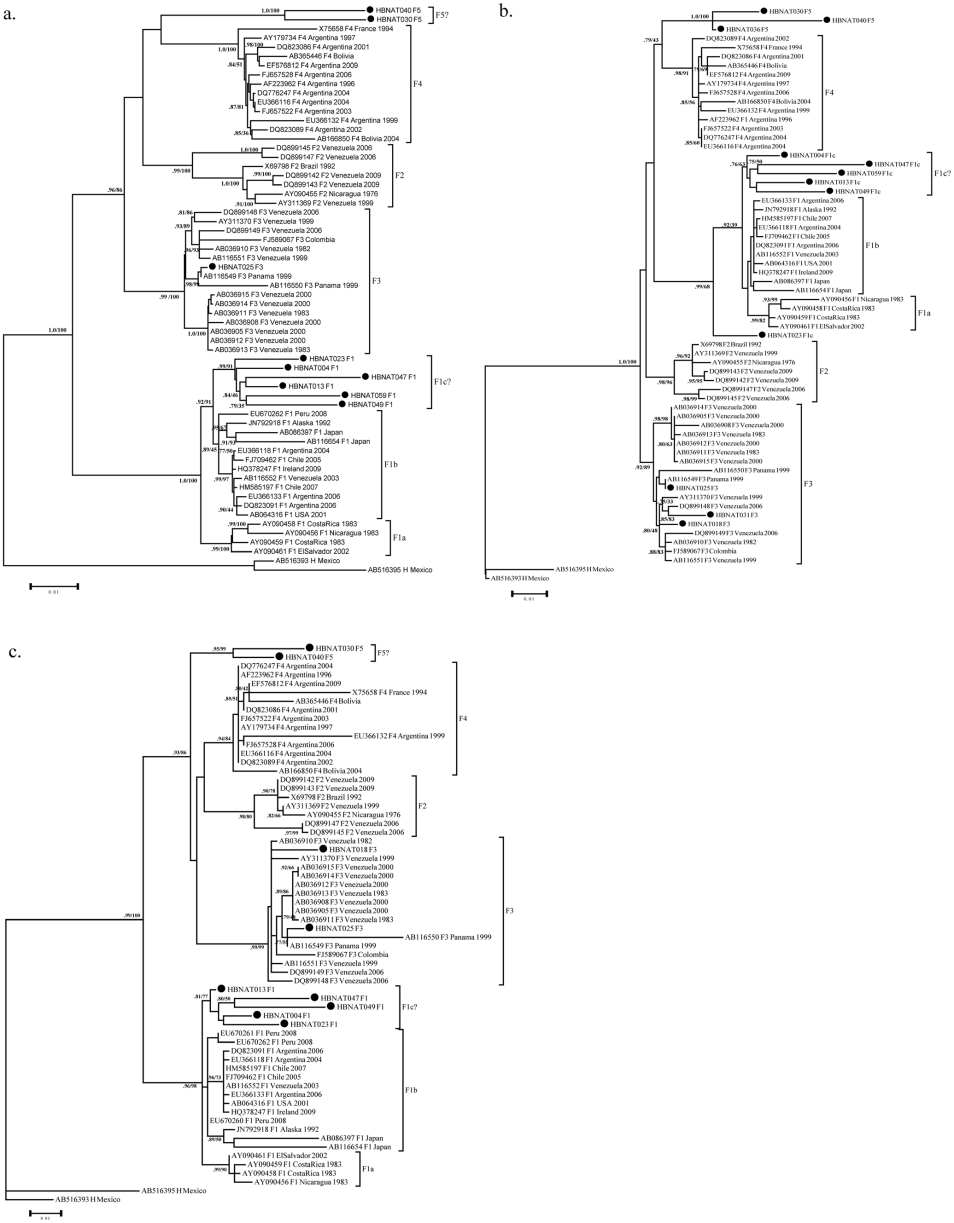
Whole genome Maximum likelihood tree constructed with HBV Genotype F sub-genotype reference sequences (F1–F4). (a), PreS2-S gene (b) and Core gene (c) (n = 75) and sequences of Panamanian origin (bold circle). Support αLTR and bootstrap values (αLTR/bootstrap) higher than 0.75 are shown, HBV genotype H is used as out-group. The length of the horizontal bars indicates the number of nucleotide substitutions per site.

Sub-genotypes of genotype F were further determined using HBV whole genome sequences of the Panamanian samples clustering in genotype F1 (n = 6), and the new formed cluster (n = 2). The sequences of sub-genotype F1 circulating in Panama again form a monophyletic clade, supported by 0.99 αLTR and 91% bootstrap value. The two sequences of the new cluster remain forming a monophyletic separated cluster of the HBV-F sub-genotypes, with a high αLTR value (1.0). The phylogenetic analysis of two HBV genome regions evidences slight differences in the relationships of the proposed F1c sub-genotype in the PreS2-S region, although the Core region shows a similar tree topology as the whole genome. (Fig. 2 a–c).

The recombination analysis gave no evidence of recombination in all the sequences analyzed.

Pair-wise analysis of nucleotide diversity was performed over the complete genome of two of the sequences forming the new cluster and six sequences of the new clade of Sub-genotype F1. The mean percentage of nucleotide divergence between the new cluster found in this study and the reference HBV sub-genotypes F range between 5.35 to 7.41 (Table 1). This result supports the classification of these sequences as a new sub-genotype F5. The nucleotide diversity among the clade formed by the Panamanian F1 sequences was 2.22 to 2.71, which falls within the definition of a clade [5].

**Table 1:** Mean percentage nucleotide divergences among the whole genome of HBV sub-genotype F.

	F1a	F1b	F1c	F2	F3	F4	F5
F1a	0.82±0.13						
F1b	1.80±019	0.63±0.06					
F1c	2.71±0.22	2.22±0.18	2.50±0.19				
F2	5.49±0.39	5.55±0.35	6.38±0.37	2.10±0.17			
F3	5.13±0.39	5.17±0.34	5.89±0.36	4.13±0.30	1.20±0.90		
F4	5.35±0.43	5.32±0.40	6.14±0.41	4.20±0.30	3.91±0.31	1.06±0.07	
F5	**6.72±0.44**	**6.74±0.41**	**7.41±0.40**	**5.52±0.33**	**5.50±0.36**	**5.35±0.34**	2.94±0.18
H	7.80±0.49	7.54±0.47	8.27±0.46	7.91±0.44	7.21±0.43	7.84±0.45	9.07±0.51

Mean ± standard deviations from averaging over all sequence pairs between groups are shown. Sub-subgenotype F5 values are displayed in bold, genotype H is added as a reference. Analyses were conducted using p-distances as implemented in MEGA5 [21].

The amino acid characterization of PreS2-S, HBsAg, Reverse transcriptase (RT) and X gene sequences showed six unique amino acids for the proposed sub-genotype F5: 124T and 172V in PreS2-S, 157M, 163V and 231L in RT protein and 44P in X protein (Table S2).

**Figure 3:**
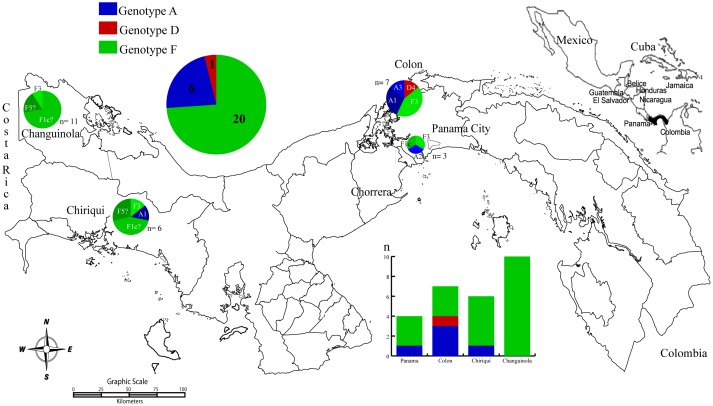
Map of Panama indicating the origin of the HBV sequences analyzed in this study. The top left pie chart indicates the number of sequences according to the genotype, the middle bar plot indicates the number of genotypes according to geographic area. The pie charts within the map indicated the proportion of the sub-genotype found in the corresponding region.

The geographic location of the HBV sub-genotypes was diverse (Fig. 3). In the most populated provinces of the country, Panama and Colon, the genotypes most frequently found were A1 (n = 3, 30%), A2 (n = 1, 10%) and F3 (n = 4, 40%). The four sequences that form the new sub-genotype F5 cluster and 8 sequences (89%) of sub-genotype F1, were geographically located in the most western area of the country: provinces of Chiriqui and Bocas del Toro (in the district of Changuinola).

Because of the low prevalence of HBeAg positive samples (n = 2, 3.5%) in Panamanian blood donors, the Basic Core promoter and PreCore mutations were evaluated. Nine sequences of Genotype F were analyzed; mutations 1762T and 1764A were found in six and seven sequences, respectively, and mutation 1896A in 6 of 8 sequences.

### Old population history of HBV genotype F observed in Panamanian samples

We employed a Bayesian framework to evaluate the time of the most recent common ancestor (tMRCA) of the Panamanian samples. According to the substitution rate used (fixed or calculated), there were differences in the model supported by the bayes factor analysis. The BSP model was chosen as the best-fit demographic model, when the time-stamped dataset was used, however, the constant Size model was chosen when fixed substitution rates were applied, (Table S3). This result reflects the flexibility of the BSP model to fit a wide range of demographic scenarios [26]. Although the Bayes factor test supports the constant size model against an exponential model in fixed substitution rates, it agrees with the epidemiological information showing that new HBV infections have remained constant in the last years in Panama [28].

The tMRCA estimates of the whole genome for the genotype F root (fixed rate: 3091 years, 2041–4554 95% HPD; time-stamped data: 633 years, 199–2143 95% HPD) were 834 years younger for the fixed dataset and, 140 younger for the time-stamped dataset. This was compared with the polymerase gene tMRCA estimates (fixed rate: 3925 years, 2148–6827 95% HPD; time-stamped data: 493 years, 99–3192 95% HPD). Additionally, there was overlapping in the 95% HPD estimates.

The tMRCA analysis of the fixed dataset shows a long history of HBV genotype F in the Americas, but with minor differences among HBV-F sub-genotypes, (Table 2). The Panamanian F1 sequences (F1pan, Table 2) have a tMRCA of 703 years (438–1034 95% HPD); the new proposed sub-genotype F (F5pan, Table 2) has a tMRCA of 845 years (366–1455 95% HPD). Compared with the rest of sub-genotype F sequences, the sub-genotype F2 shows the oldest tMRCA. When the F1c and F5 sequences were compared with the sub-genotypes F1a, F1b, F3, F4, the Panamanian sequences (F1pan, F5pan) have a slightly older tMRCA. However, the 95% HPD estimates for these sub-genotypes show considerable overlap.

**Table 2:** Estimated of tMRCA for Genotype F, with Whole genome, polymerase and PreC-Core fragments of Panamanian samples.

	Group	Whole Genome (Partitioned model)		Polymerase	
Substitution Rate		tMRCA (years)	95% HPD interval (years)	tMRCA (years)	95% HPD interval (years)
	F1a	372	155–688	375	154–692
	F1b	570	314–897	735	319–1230
	F1pan	703	438–1034	699	378–1091
Fixed (1.5 E-5)	F2	1190	741–1806	1190	681–1865
	F3	656	406–1001	1061	659–1597
	F3pan	622	369–931	1035	620–1558
	F4	634	311–1094	763	364–1317
	F5pan	845	366–1455	966	446–1684
	F1a	83	37–234	78	32–412
	F1b	104	43–342	108	22–664
	F1pan	101	37–376	84	14–499
	F2	216	76–701	203	49–1288
Time stamped-data	F3	119	50–388	153	39–934
	F3pan	113	46–366	148	37–903
	F4	118	39–393	117	23–697
	F5pan	120	27–477	148	13–1038
	Mean rate*	8.9×10^−5^	1.3×10^−5^ 1.7×10^−4^	1.04×10^−4^	1.8×10^−7^ 2.2×10^−4^

tMRCA calculated by calibration with a fixed substitution mean rate (1.5 E-5) and time-stamped data under the best-fit demographic model indicated by the log-marginal likelihood analysis (appendix, S3). The Bayesian analysis was performed for the HBV whole genome, and a portion (876 pb) of polymerase gene from the available sequences (databases available upon request). The years correspond to dates in the past.

*substitutions/site/year.

**Figure 4:**
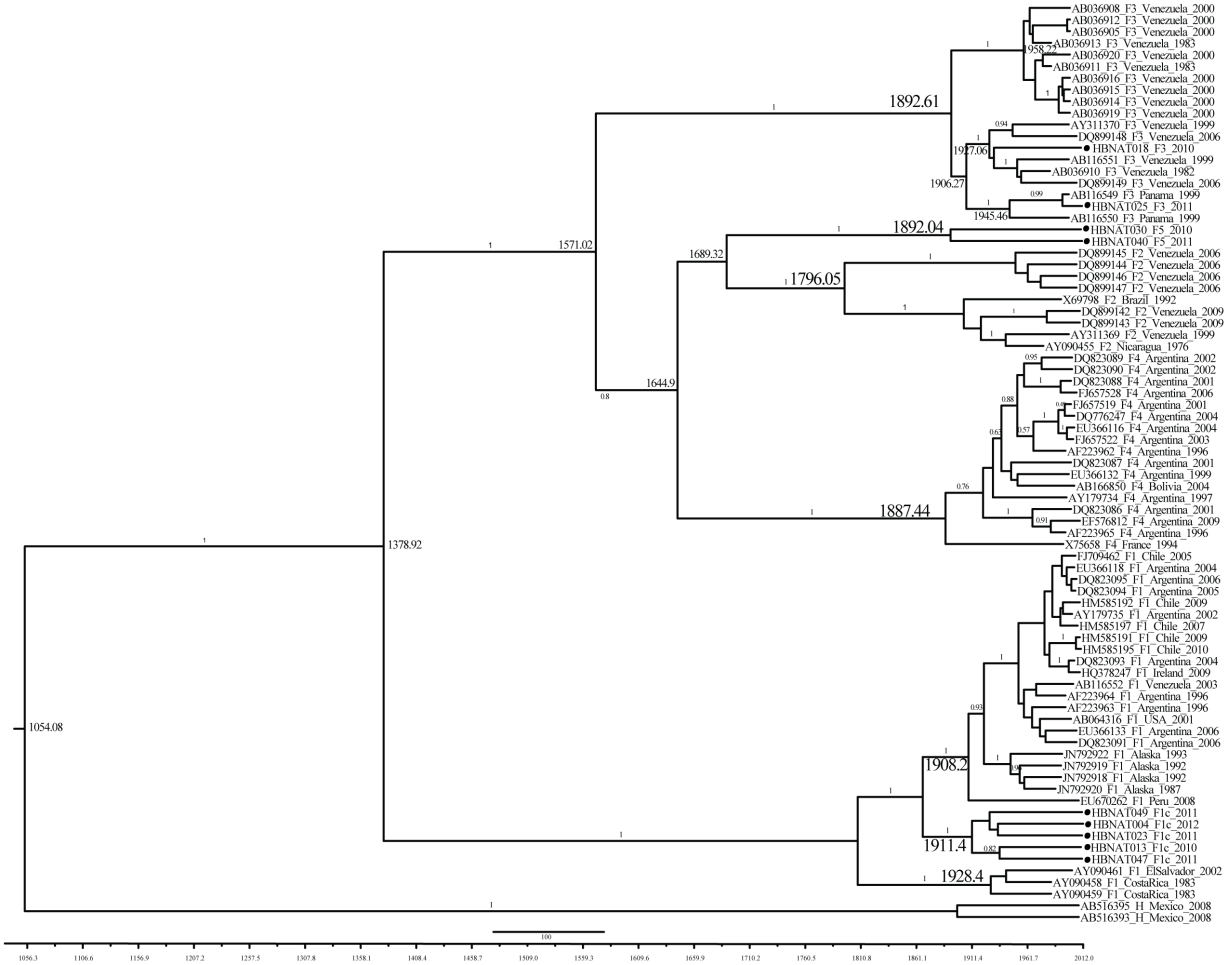
Time resolved phylogenetic tree showing the relationships among the whole genome sequences of Panamanian samples and sub-genotypes F of diverse origin. The date of MRCA is shown in the node of relevant sub-genotypes and nodes formed by Panamanian samples. Bayesian posterior probabilities (>0.75) of each clade is shown above the relevant branch.

The estimates of tMRCA with the time-stamped dataset show a more recent history of HBV genotype F in the Americas. The tMRCA for Panamanian sequences (F1pan, F5pan) fall between the period of 1892 to 1911 (Fig 4). The oldest sub-genotype was the F2 of 1796, followed by F4 of 1887, and F3 of 1892; these have a tMRCA similar to the F5pan sequences.

## Discussion

The diversity of HBV genotypes in Panama is greater than expected (Fig. 1). We determined circulation of three major genotypes (A, D, F), and at least five previously described sub-genotypes (A1, A2, A3, F1pan, F3). Interestingly, a new diversification of genotype F cluster was also observed in the phylogenetic tree analysis (see Fig. 1, bottom). This new cluster is entirely composed by strains isolated from Panama. Since this new cluster has a significantly high statistical support by the values of αLRT and bootstrap obtained, we propose a new sub-genotype, F5pan. This result overrides previous reports, which indicated only the circulation of genotype F3 in the Panamanian population [16], [29].

The results of the study are also in agreement with the observed migration of distinct genotypes across the globe. For example, genotype A1 was recently traced in the Americas [8], it was reported in an Afro-descendant population of Colombia [30] and in several regions of Brazil [31]–[33]. Sub-genotype A2 has been recently linked to high-risk groups: in Argentina to drug users [34] and men that have sex with men (MSM) [35], and in Japan to MSM groups [36]. Because of the experimental design of this study, we did not have the opportunity to evaluate risk behaviors of HBV infected blood donors. More studies are needed to address this important issue. In addition, studies intended to evaluate the HBV prevalence and genotypes circulation in specific groups: female sex workers (FSW), MSM and others are missing. Additionally, the only sub-genotype A3 sample circulating in blood donors was closely related to sequences of Haitian origin.

The genotype F was the most diverse HBV genotype in Panama. This genotype includes a monophyletic clade (F1c?), which is separated from the previous reported F1a and F1b clades, and that has a relative high divergence among members (p-distance  = 2.50±0.19). This diversity most likely results from a long circulation history of these sub-genotypes (F1c) in Panama. Interestingly, the fact that almost all the F1c samples came from the northwestern region of Panama (Chiriqui province and Changuinola district) indicates a probable historical presence of this virus in Panama.

Additionally, we have observed that a group of four genotype F sequences forms a monophyletic cluster with a high support value (αLTR  = 1.0, bootstrap  = 100%), separated from the rest of HBV sub-genotype F sequences. The molecular analysis of the complete genome of these sequences evidences a nucleotide divergence greater than 4 percent, when compared with the rest of available HBV-F sub-genotypes sequences. Furthermore, the four sequences have specific amino acids changes in preS2-S, X and polymerase proteins. These results fulfill the requirements for the designation of this group of sequences as a novel HBV sub-genotype F (F5) [6]. Sequences of sub-genotype F5 and F1c were located in the same geographical area of Panama, despite the existence of a great genetic distance between them (p-distance  = 7.41±0.40). This probably suggests different introductions of these variants in the region.

A Bayesian approach was used to estimate the population history of the Panamanian HBV-F sub-genotypes (F1pan, F5pan). These sub-genotypes have an estimated time of most common ancestor similar to the rest of the HBV-F sub-genotypes (F1–F4). The high variability of the HBV genotypes and its distinctive distribution across the continent, imply a long history of migration and isolation of the common ancestors of the sub-genotypes currently circulating in the Americas. The results of this study suggest that the common ancestors of the Panamanian sub-genotypes (F1pan, F5pan) were part of the diversification history of the genotype F in the Americas, regardless of the tree priors used (fixed substitution rate or time-stamped data) or the genome region employed (whole genome, polymerase gene) to perform the analysis.

Another important result is the evidence of the confinement of these genotypes (F1pan, F5pan) in the most western region of Panama. This region is characterized by a high incidence of HBV infection among adults.

It has been hypothesized that the HBV sub-genotypes F sequences described thus far, do not represent the complete history of HBV genotypes in the Americas. The tMRCA estimated for sub-genotype F Panamanian sequences confirm this hypothesis. The results of this study suggest a simultaneous circulation of the Panamanian samples with previously described sub-genotypes, rather than a recent origin of the Panamanian sub-genotypes in the country.

In conclusion, Panama has a high HBV genotype diversity that includes a new proposed sub-genotype, an elevated number of PreCore-Core mutations, and confinement of these variants to a specific geographical region. These results emphasize the importance to study the molecular epidemiology of HBV in Central America.

## Supporting Information

Table S1 **Partitions of the HBV Whole genomes.** Partitions of the HBV Whole genomes and the corresponding substitution model used in the Bayesian analysis. Abbreviations HYY: Hasegawa-Kishino-Yano model; GTR: Generalized time reversible model; SYM: symmetrical model.
(DOCX)

Table S2 **Multiple alignment of the deduced amino acid substitutions in PreS-S region, X gene, Pol/RT proteins.** Panamanian samples name are in bold. Only position with unique substitutions are shown, dots indicate the same amino acid as reference, - indicates no sequence available. Position highlighted in gray have unique substitutions for the proposed sub-genotype F5. * No X-gene sequence available for these isolates.
(XLSX)

Table S3 **Model selection using maximum likelihood estimates to calculate the path Sampling (PS) and stepping stone sampling (SS) estimates.** The best-fit model (bold) was determined using the log-marginal likelihood of each grow model, with a fixed substitution rate (1.5×10^−5^ subst/site/year) or using time-stamped data to estimate the substitution rates. The Constant size model is the null model used in the Bayes factor test. CS: Constant size grow model, Expo: Exponential grow model, BSP: Bayesian skyline plot model.
(DOCX)
