# Supplementary material for: High Diversity of Hepatitis B Virus Genotypes in Panamanian Blood Donors: A Molecular Analysis of New Variants
Source: PLoS One. 2014 Aug 5;9(8):e103545. doi: 10.1371/journal.pone.0103545 (PMC4122375; doi:10.1371/journal.pone.0103545)
Supplement: Table S1 — Partitions of the HBV Whole genomes. Partitions of the HBV Whole genomes and the corresponding substitution model used in the Bayesian analysis. Abbreviations HYY: Hasegawa-Kishino-Yano model; GTR: Generalized time reversible model; SYM: symmetrical model. (DOCX) [file pone.0103545.s001.docx]

**Table S1** Partitions of the HBV Whole genomes

| **Region name** | **nucleotides** | **Substitution model** |
| --- | --- | --- |
| S2-pol | 1 – 255 | HKY |
| HBsAg-pol2 | 256 – 835 | GTR |
| Pol Non-overlapping | 836 – 1373 | SYM |
| Pol X-Gene | 1374 – 1623 | GTR |
| X-Gene-Pre-Core Non-overlapping | 1624 – 2306 | HKY |
| Core-Pol | 2307 – 2452 | HKY |
| Pol Non-overlapping | 2453 – 2847 | HKY |
| Pol-PreS | 2848 – 3215 | HKY |

Partitions of the HBV Whole genomes and the corresponding substitution model used in the Bayesian analysis. Abbreviations HYY: Hasegawa-Kishino-Yano model; GTR: Generalized time reversible model; SYM: symmetrical model
