# Supplementary material for: High Diversity of Hepatitis B Virus Genotypes in Panamanian Blood Donors: A Molecular Analysis of New Variants
Source: PLoS One. 2014 Aug 5;9(8):e103545. doi: 10.1371/journal.pone.0103545 (PMC4122375; doi:10.1371/journal.pone.0103545)
Supplement: Table S3 — Model selection using maximum likelihood estimates to calculate the path Sampling (PS) and stepping stone sampling (SS) estimates. The best-fit model (bold) was determined using the log-marginal likelihood of each grow model, with a fixed substitution rate (1.5×10^−5^ subst/site/year) or using time-stamped data to estimate the substitution rates. The Constant size model is the null model used in the Bayes factor test. CS: Constant size grow model, Expo: Exponential grow model, BSP: Bayesian skyline plot model. (DOCX) [file pone.0103545.s003.docx]

**Table S3** Model selection using maximum likelihood estimates to calculate the path Sampling (PS) and stepping stone sampling (SS) estimates.

|  | | **Path Sampling** | | | |  | **Stepping Stone** | | | |  |
| --- | --- | --- | --- | --- | --- | --- | --- | --- | --- | --- | --- |
|  | | Constant size (CS) | Exponential Grow (Expo) | **Log BF CS/Expo** | Bayesian skyline (BSP) | **Log BF CS/BSP** | Constant size (CS) | Exponential Grow (Expo) | **Log BF CS/Expo** | Bayesian skyline (BSP) | **Log BF CS/BSP** |
| **Whole genome** | fixed | ***-15809.0*** | -15807.2 | -1.79 | -15804.8 | -4.21 | -15809.6 | ***-15811.68*** | 2.08 | -15807.1 | -2.49 |
|  | time-stamped | -15744.8 | -15720.23 | -24.59 | **-15766.5** | 21.64 | -15742.8 | -15719.59 | -23.27 | **-15763.5** | 20.67 |
| Log fixed / time-stamped | | -64.2 | -86.97 |  | -38.3 |  | -66.8 | -92.09 |  | -43.6 |  |
| **Polymerase** | fixed | ***-4275.98*** | -4270.87 | -5.11 | -4258.77 | -17.21 | ***-4275.71*** | -4273.26 | -2.45 | -4260.16 | -15.55 |
|  | time-stamped | -4271.01 | -4269.60 | -1.41 | **-4272.36** | 1.35 | -4270.51 | -4271.35 | -0.84 | **-4273.88** | 3.37 |
| Log fixed/time-stamped | | -4.97 | -1.27 |  | 13.59 |  | -5.2 | -1.91 |  | 13.72 |  |

The best-fit model (bold) was determined using the log-marginal likelihood of each grow model, with a fixed substitution rate (1.5 X 10^-5^ subst/site/year) or using time-stamped data to estimate the substitution rates. The Constant size model is the null model used in the Bayes factor test. CS: Constant size grow model, Expo: Exponential grow model, BSP: Bayesian skyline plot model.
